# A Woman with Four Breasts

**Published:** 1870-11

**Authors:** 


### A Woman with Four Breasts.

A primiparous woman was admitted under M. Lorain, and was delivered next day of a dead premature child. She was found to have four breasts, two in the normal position, and with the normal puerperal appearances, and two which, from their position, might be called axillary, and attaining the size of a small orange. She menstruated at twelve, and at the periods she experienced pain in the small breasts. The colostrum also, which these contained, was small in quantity, and the granular bodies were less and transparent, while the milk-globules were fewer. The areolæ was also very small. In spite of an attack of fever, the lacteal secretion was regularly established in all the breasts, but the milk examined microscopically was found of a much poorer quality in the supplementary breasts.—*Revue Photographique des Hopitaux.*—*New York Medical Journal.*

